# Syphilis and Tuberculosis as Mimickers of Autoimmune Diseases: Diagnostic Overlap and Surveillance Implications in Mexico

**DOI:** 10.3390/diseases13100318

**Published:** 2025-09-28

**Authors:** Gustavo Esteban Lugo-Zamudio, Oscar Sosa-Hernández, Briceida López-Martínez, Clemente Cruz-Cruz, Emilio Mariano Durán-Manuel, Miguel Ángel Loyola-Cruz, José Carlos Gasca-Aldama, Paulina Carpinteyro-Espin, Luis Gustavo Zárate-Sánchez, Enzo Vásquez-Jiménez, Juan Manuel Bello-López

**Affiliations:** 1 Dirección General, Hospital Juárez de México, Mexico City 07760, Mexico; 2 División de Calidad de la Atención, Hospital Juárez de México, Mexico City 07760, Mexico; 3 Laboratorio Clínico, Hospital Juárez de México, Mexico City 07760, Mexico; 4 División de Investigación, Hospital Juárez de México, Mexico City 07760, Mexico; 5 Unidad de Cuidados Intensivos, Hospital Juárez de México, Mexico City 07760, Mexico; 6 Departamento de Trasplantes, Hospital Juárez de México, Mexico City 07760, Mexico; 7 División de Vinculación y Seguimiento Clinico, Hospital Juárez de México, Mexico City 07760, Mexico; 8 Servicio de Nefrología, Hospital Juárez de México, Mexico City 07760, Mexico

**Keywords:** syphilis, tuberculosis, epidemiological surveillance, autoimmune diseases

## Abstract

In Mexico, syphilis and tuberculosis are infectious diseases subject to mandatory and immediate epidemiological surveillance, both with special systems that allow nominal follow-up for either variant. Surveillance uses the operational definitions of probable and confirmed cases established in the manual for epidemiological surveillance issued by the General Directorate of Epidemiology of the Ministry of Health. However, both diseases, mainly in the chronic state, present challenges because of their ability to mimic autoimmune disorders. This review explores the phenomenon of clinical and immunological mimicry in secondary and tertiary syphilis, as well as in extrapulmonary tuberculosis, and analyzes its implications for the accuracy of case reporting at the national level. Evidence shows that both infections can present systemic inflammatory features, such as elevated acute phase reactants, positive autoantibodies, and alterations in cerebrospinal fluid that resemble autoimmune profiles. These overlaps can lead to misdiagnosis, inappropriate immunosuppressive treatment and misclassification of confirmed cases within the Mexican surveillance system. Surveillance of these conditions is robust; however, current operational definitions have weaknesses, particularly when atypical or autoimmune conditions are present, as they only focus on cases with the highest prevalence or public health impact. This manuscript proposes the integration of differential diagnostic algorithms and expanded laboratory criteria, including autoimmune markers and molecular tests, into surveillance protocols. Although individual efforts exist in health institutions, in our country, the absence of autoimmune diseases in the national register of obligatory notification stands out, contrasting with surveillance models in other countries, where autoimmune diseases are tracked systematically. To improve diagnostic accuracy and reporting, surveillance systems should incorporate a syndromic and etiological approach, recognizing infectious autoimmune mimicry as a factor in the final recording of confirmed cases to avoid epidemiological silence.

## 1. Introduction

Epidemiological surveillance in all countries of the world constitutes one of the fundamental requirements for the timely detection, notification, and control of communicable diseases; undoubtedly, the COVID-19 pandemic was a clear example of its usefulness, demonstrating, in some cases, its collapse in the face of other diseases and the need for this surveillance to be strengthened [1,2,3,4]. In Mexico, this surveillance is governed by the Official Mexican Standard NOM-017-SSA2-2012 “for epidemiological surveillance” which establishes the criteria, specifications, and guidelines for the operation of the National Epidemiological Surveillance System (or SINAVE, its acronym in Spanish) in Mexico and by the *Manual of Standardized Procedures for the Epidemiological Surveillance of Notifiable Diseases*, which establishes guidelines and protocols for the collection and analysis of information on diseases whose notification is mandatory by law and which establishes operational definitions of probable and confirmed cases for each disease [5].

In the case of infectious diseases, such as syphilis and tuberculosis, both are included in this system, both for weekly notification and for nominal follow-up through special epidemiological surveillance systems, due to their public health relevance and the clinical implications associated with their progression. Such is their relevance in the national context that several epidemiological investigations in our working group have documented the dynamics of these two diseases in terms of their geographical distribution, incidence, and cumulative confirmed cases. Likewise, the most vulnerable age groups have been identified, and, in some cases, projections have been made to estimate their epidemiological behaviour in the coming years [6,7].

In Mexico, both syphilis and tuberculosis are subject to epidemiological surveillance. In the case of tuberculosis, there is a special system and the *Standardized Procedures Manual for the Epidemiological Surveillance of Mycobacteriosis*, which establishes the timing of notification according to the classification of tuberculosis. In the case of syphilis, for congenital syphilis, there is the *Manual of Standardized Procedures for Epidemiological Surveillance of Congenital Syphilis*, while for other forms of syphilis there is the *Manual of Standardized Procedures for Epidemiological Surveillance of Notifiable Diseases* [8]. These manuals establish operational definitions with broad sensitivity to classify cases as suspected, probable, confirmed, or ruled out, based on clinical features and laboratory findings, to ensure systematic detection throughout the country.

Although these manuals represent an important advance in disease detection, prevention, and control, they also show certain limitations, as they may leave out clinical and laboratory presentations that do not follow the typical pattern of these diseases. In this context, in routine medical practice, not all patients present with typical symptoms and signs, and laboratory tests may be inconclusive, where the system can fail when an infection could be mistaken for another pathology of a completely different nature. An under-recognized and underexplored phenomenon in this context is “mimicry”, i.e., when infectious diseases, such as syphilis or tuberculosis, take on forms that resemble autoimmune disorders [9,10,11,12].

This ability to “mask” can confuse even the most experienced medical staff, delay diagnoses, hinder the proper application of criteria by the operational definition’s manual, and, consequently, lead to over- or under-reporting of cases. This overlap has been historically documented in the national and international medical literature.

Historically, cases of secondary syphilis mimicking diseases, such as systemic lupus erythematosus, vasculitis, or systemic sclerosis, have been reported [13,14,15]. Similarly, there are extrapulmonary forms of tuberculosis that may resemble rheumatological, neurological, or dermatological pictures of autoimmune origin [16,17,18,19,20]. Under these scenarios, criteria focused only on detecting specific infections may not be sufficient, and a broader view is needed in order not to lose sight of alternate diagnoses, as although these diseases have different etiopathogenic diagnoses, the clinical pictures may be indistinguishable with autoimmune diseases. Therefore, this manuscript provides a critical review of the strengths and weaknesses of the operational definitions used in Mexico, particularly for syphilis and tuberculosis, which can mimic autoimmune conditions, and raises the urgent need to revise and enrich these definitions to improve the criteria for the detection of confirmed cases in epidemiological surveillance systems. It also reflects on the omission of autoimmune diseases from the SINAVE, which limits the possibility of establishing a national epidemiological profile and, thus, establishing a true differential diagnosis from a simultaneous “infectious disease” and “autoimmune disease” perspective.

## 2. Strengths of Epidemiological Surveillance Based on Operational Definitions

Mexico’s SINAVE has proven to have a robust structure, capable of good detection of infectious, chronic, and non-communicable diseases, thanks to the systematic use of operational definitions of suspected, probable, confirmed and excluded cases. These operational definitions are based on international standards, such as the World Health Organization (WHO), Pan American Health Organization (PAHO) and in some cases by world reference centers, such as the Centers for Disease Control and Prevention (CDC) in the United States. These definitions make it possible to establish standardized criteria at the national level for the identification of cases, which guarantees uniformity in the notification to the General Directorate of Epidemiology (DGE), as well as the monitoring of priority diseases in public health.

The information generated through this system has allowed the development of retrospective studies with great scientific value, which have not only contributed to understanding epidemiological trends, but have also provided useful evidence for decision-making on treatment, prevention, and control, especially in vulnerable populations [6,7,21,22,23,24,25,26]. This work has demonstrated the system’s ability to generate comparable epidemiological trends over time, across regions and, in some cases, across countries. This quality makes it possible to evaluate the effectiveness of public health interventions, adjust health policies and even prioritize resources more efficiently. In contrast, operational definitions have also proven to be useful tools in the diagnostic orientation of diseases considered rare or unusual in certain geographical regions, especially when they occur outside their traditional endemic areas.

This approach has made it possible to more accurately identify imported cases that might otherwise go undetected without clear diagnostic criteria. An example of this is the recent reports in our working group where we identified and reported cases of imported *Plasmodium falciparum* and *P. vivax* malaria in migrants from Africa and Latin America, where operational definitions contributed significantly to timely diagnosis and implementation of control measures [25,26]. Nevertheless, even though there are many more strengths, these become limitations when operational definitions do not take into account atypical clinical manifestations and laboratory findings or conditions that share symptomatology with other diseases, such as mimicry with autoimmune diseases.

## 3. Challenges in Operational Definitions: Mimicry with Autoimmune Diseases

**Figure 1:**
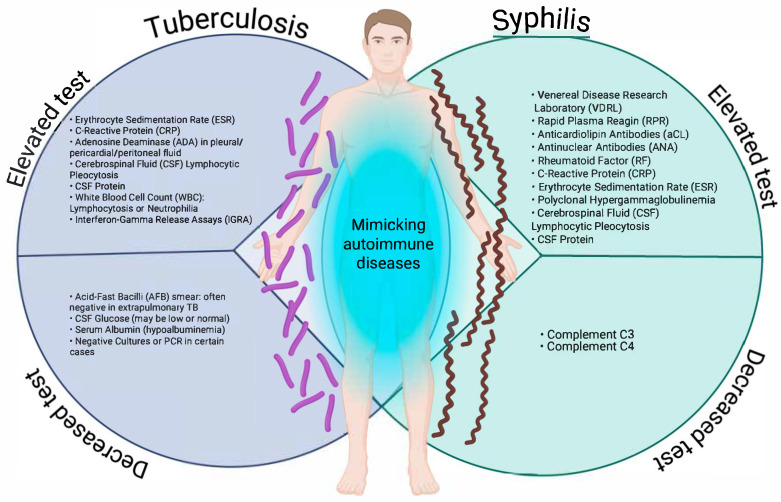
Increasing and decreasing laboratory test patterns in tuberculosis and syphilis that may mimic autoimmune diseases. Created in BioRender. https://BioRender.com/ewg2cwu.

In a state, national, or global surveillance system, the nature of operational definitions is to detect as many suspected or probable cases as possible, i.e., to be as sensitive as possible, and from these cases, according to clinical and laboratory criteria, to make the definitive diagnosis to detect confirmed or ruled out cases, which makes them non-specific; this is carried out in order to direct material and human resources according to the burden of these diseases; and this represents a weakness in the approach to infectious diseases that can manifest themselves atypically or clinically overlap with autoimmune diseases. This is particularly the case for syphilis and tuberculosis, two infectious diseases that can adopt clinical and laboratory forms that mimic autoimmune diseases, such as systemic lupus erythematosus, rheumatoid arthritis, vasculitis, antiphospholipid syndrome, or even multiple sclerosis [13,16,18,19,20,25]. Figure 1 illustrates the patterns of laboratory tests that may be altered (increased or decreased) in tuberculosis and syphilis, two infections that can mimic autoimmune diseases due to the systemic immune activation they induce.

As can be observed, both entities share alterations, such as elevated acute phase reactants (C-reactive protein (CRP), erythrocyte sedimentation rate (ESR)), lymphocytic pleocytosis, and increased protein in cerebrospinal fluid (CSF)). Particularly relevant are serological findings typically associated with autoimmunity, such as antinuclear antibodies (ANA), rheumatoid factor (RF), and anticardiolipin antibodies in secondary syphilis, which may lead to misdiagnoses, such as lupus or antiphospholipid syndrome. Conversely, extrapulmonary tuberculosis shows similar inflammatory patterns, accompanied by non-specific tests, such as interferon gamma release assay (IGRA), with decreased or normal CSF glucose, and hypoalbuminemia.

In the case of syphilis, complement proteins C3 and C4 may be decreased, indicative of an autoimmune condition [27,28,29]. The overlap of these biochemical and serological alterations, as represented, reinforces the need to consider infectious etiologies in patients with suspected autoimmune disease before initiating immunosuppressive therapy. In the clinical context, both syphilis and tuberculosis can present with multisystemic manifestations that mimic autoimmune diseases, making timely diagnosis difficult. This phenomenon, known as immunological mimicry, is mainly observed in secondary syphilis and extrapulmonary tuberculosis, infectious forms that induce intense immune activation and generate symptoms similar to systemic lupus erythematosus, vasculitis, Sjögren’s syndrome, spondyloarthropathies, or sarcoidosis. Table 1 summarises the main clinical manifestations of these infections that can lead to misdiagnosis by mimicking autoimmune diseases.

In the case of secondary syphilis, palmoplantar rash, patchy alopecia, Raynaud’s phenomenon, and non-specific neurological symptoms, such as lymphocytic meningitis, which share similarities with the typical findings of lupus or systemic sclerosis, stand out. Extrapulmonary tuberculosis may manifest with fever of unknown origin, persistent lymphadenopathy, sacroiliitis, or meningeal involvement, which resembles diseases, such as sarcoidosis or neuropsychiatric lupus. It should also be recognized that tuberculosis may mimic not only autoimmune diseases but also certain malignancies. For instance, intestinal tuberculosis is often misdiagnosed as colorectal cancer, while tuberculous meningitis may resemble glioblastoma. Although these conditions represent well-documented diagnostic challenges, our review deliberately focused on autoimmune overlap because it is rarely considered in the current operational definitions of epidemiological surveillance. Nevertheless, acknowledging malignancies as part of the differential diagnosis underscores the broad spectrum of tuberculosis mimics and the importance of comprehensive diagnostic algorithms [38,39]. These similarities may lead to the misuse of immunosuppressants if infectious causes are not ruled out by specific serological and microbiological tests, which could even include molecular testing in the search for specific genetic fingerprints of the pathogen [40,41,42,43,44].

These situations reflect a weakness in the operational definitions as they do not contemplate algorithms to rule out infectious etiologies when the clinical picture resembles an autoimmune disease. Therefore, modification of confirmed case definitions to include differential diagnoses is necessary to improve surveillance and management of both diseases. In addition, from the side of rheumatology care, it is also possible to confirm or rule out such conditions as tuberculosis, since, in turn, due to the characteristics of the treatments, it is necessary to clarify negative results in order not to complicate the health of patients.

Several case reports illustrate how syphilis and tuberculosis can imitate autoimmune disorders, sometimes leading to misdiagnosis and inappropriate treatment. In secondary syphilis, for instance, clinical features have been mistaken for systemic lupus erythematosus or rheumatoid arthritis [13,14]. Cases of neurosyphilis have also been described where the presentation resembled connective tissue diseases [11,12,32]. Extrapulmonary tuberculosis shows a similar pattern: it may appear as Poncet’s disease (reactive arthritis) or as cutaneous and pulmonary forms that resemble rheumatological or dermatological autoimmune conditions [16,17,19,20]. These reports highlight a recurring diagnostic challenge. Importantly, there are not only isolated cases. Systematic reviews and meta-analyses have documented the same problem. Li and Wang (2022) underscored the difficulty of distinguishing tuberculosis from autoimmune diseases in daily clinical practice [29]. Another meta-analysis showed the limited specificity of interferon-gamma release assays in cerebrospinal fluid when used to diagnose tuberculous meningitis, a condition that often mimics autoimmune neurological disorders [33].

Recent reviews also emphasize syphilis as a frequent imitator of autoimmune disease, particularly neurosyphilis with its wide clinical spectrum [31,32]. In parallel, comprehensive analyses of tuberculosis reaffirm its ability to resemble systemic conditions, such as sarcoidosis or spondyloarthropathies [34]. Taken together, this evidence strengthens the case for expanding operational definitions to include differential diagnoses that account for infectious–autoimmune mimicry.

## 4. Biological Basis of Mimicry: Syphilis and Tuberculosis

Mimicry between infectious and autoimmune diseases can be explained by biological mechanisms involving dysregulated host immune activation, the production of autoantibodies, and molecular interaction between the pathogen and the immune system. Both *T. pallidum* and *M. tuberculosis* induce complex immune responses that can lead to clinical pictures similar to those seen in autoimmune diseases.

### 4.1. Syphilis and Autoimmunity

Secondary and tertiary syphilis can generate dermatological, neurological, and articular clinical manifestations that are easily confused with connective tissue diseases. At the immunological level, it has been shown that *T. pallidum* infection can induce the production of autoantibodies, such as ANA, rheumatoid factor, and anti-phospholipid antibodies. This response may be transient or persistent, which contributes to the diagnostic overlap with entities, such as systemic lupus erythematosus, rheumatoid arthritis, or antiphospholipid syndrome. Furthermore, *T. pallidum* spirochetes can evade the immune system through antigenic variations and modulation of the T-cell response, leading to chronic inflammation and prolonged or atypical clinical pictures.

**Figure 2:**
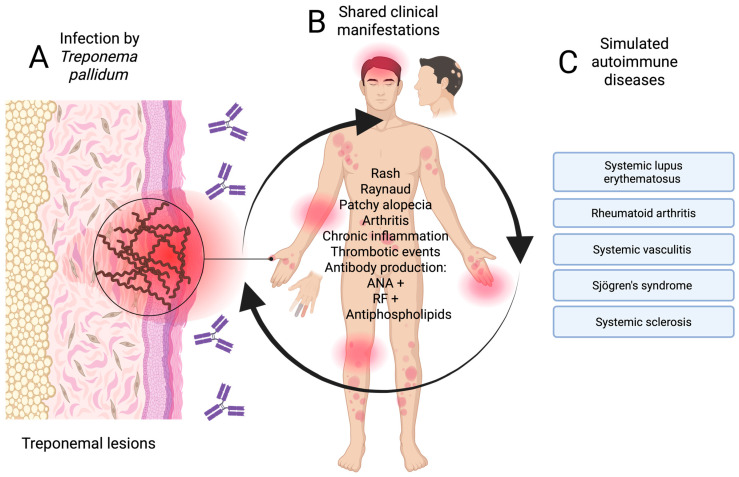
Pathway of syphilis mimicry (secondary and tertiary) in autoimmune diseases. (**A**) Infection stage, (**B**) atypical clinical manifestations, and (**C**) mimicking autoimmune diseases. Created in BioRender. https://BioRender.com/3w4jcno.

In patients with latent or tertiary syphilis, neurological (neurosyphilis), articular, or cardiovascular manifestations may be confused with systemic vasculitis or demyelinating diseases, such as multiple sclerosis [45,46,47]. Figure 2 summarises the pathways that give rise to immunological mimicry of mainly secondary and tertiary syphilis with diseases of autoimmune etiology.

### 4.2. Extrapulmonary Tuberculosis: Immune Activation and Simulated Rheumatological Pictures

Extrapulmonary tuberculosis presents a diagnostic challenge when it manifests as fever of unknown origin, polyarthritis, serositis, or neurological symptoms. In Poncet’s disease, for example, non-suppurative reactive arthritis occurs in the context of active tuberculosis, without evidence of bacilli, which can mimic juvenile idiopathic arthritis or seronegative rheumatoid arthritis [16]. The immunopathological mechanisms include T-cell-mediated type IV hypersensitivity, the release of proinflammatory cytokines, such as TNF-α and IFN-γ, and cross-reactivity between mycobacterial antigens and host self-antigens. In these patients, elevated titers of ANA, ESR, CRP, and other biomarkers of inflammation have also been documented, which may lead to misdiagnosis of a primary autoimmune disease [17,48].

**Figure 3:**
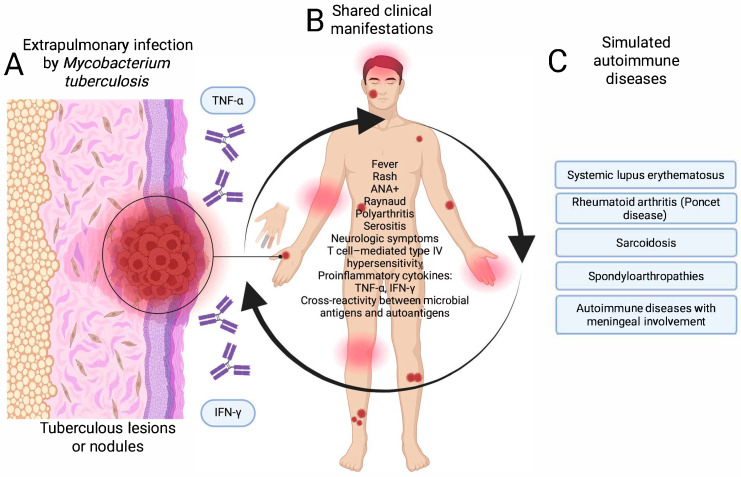
Pathway of mimicry of extrapulmonary tuberculosis with autoimmune diseases. (**A**) Infection stage, (**B**) atypical clinical manifestations, and (**C**) mimicking autoimmune diseases. Created in BioRender. https://BioRender.com/ptz1lve.

In both cases, activation of the immune system and production of autoantibodies in the absence of an adequate clinical and microbiological context can lead to diagnostic errors and counterproductive therapeutic decisions, such as the use of immunosuppressants. Figure 3 shows the pathways of mimicry of extrapulmonary tuberculosis with diseases of autoimmune etiology.

## 5. Modification of Operational Definitions for Syphilis and Tuberculosis

Given the risk of potential under-reporting of confirmed cases of infectious diseases that could mimic autoimmune pathologies, it is essential to review and update the operational definitions of confirmed cases in the *Mexican Epidemiological Surveillance Manual*. The current definitions, while useful in conventional settings, are insufficient when the clinical presentation is atypical. The integration of differential algorithms in the operational definitions could be used to differentiate infectious diseases, such as syphilis and tuberculosis, from autoimmune diseases. This would involve the use of markers, such as ANA, RF, antiphospholipid antibodies, and acute phase reactants (ESR and CRP).

It should be noted that serological tests in syphilis may generate false positive results in patients with autoimmune diseases, which may reduce clinical mimicry. Alternatively, treponemal tests using direct measurement methods, such as darkfield microscopy or PCR, may be more useful in early stages of the disease [49]. In addition, integrating nucleic acid testing into confirmed case definitions for other non-conventional clinical forms, such as neurosyphilis or extrapulmonary tuberculosis, may be useful. Undoubtedly, these modifications should be accompanied by continuous training of medical and epidemiological surveillance personnel in the detection of infectious diseases with immunological mimicry.

To a certain extent, it is understandable and justifiable that the current operational definitions respond to the need to cover the greatest volume of patients due to the impact that these diseases have on public health in our country, but it is also important to address special cases, allowing the opening of the evaluation of results in special cases or cases different from the conventional ones, in addition to also considering the opening of access to targeted treatments. Beyond revising operational definitions, practical tools are needed to minimize misclassification. Several novel diagnostic approaches could be incorporated into future surveillance protocols. In tuberculosis, classical tools, such as sputum microscopy, chest radiography, and MRI, remain important, while more advanced tests, like GeneXpert MTB/RIF, and interferon-gamma release assays (e.g., Quantiferon TB Gold) provide higher specificity [50,51]. Beyond conventional diagnostic approaches, interferon-gamma release assays (IGRAs), such as Quantiferon TB Gold, have been increasingly recognized as valuable tools in tuberculosis, not only for diagnosis but also with potential prognostic implications. Previous reports highlight that IFN-γ responses may reflect disease activity and treatment response, particularly in extrapulmonary and paucibacillary forms of tuberculosis [29,34]. Although these assays are not widely integrated into epidemiological surveillance in Mexico, their incorporation into differential diagnostic protocols could improve specificity and provide insights into therapeutic monitoring. Nevertheless, even with these tools, diagnostic overlap can persist in extrapulmonary or paucibacillary disease, underscoring the need to integrate clinical, immunological, and molecular criteria into surveillance protocols.

In neurosyphilis, the use of cerebrospinal fluid PCR targeting *T. pallidum* genes has shown higher specificity than conventional serology, while CXCL13 levels in CSF have been proposed as a biomarker of intrathecal immune activation [38,52]. For tuberculosis, nucleic acid amplification tests and urine lipoarabinomannan (LAM) detection represent promising strategies for extrapulmonary and paucibacillary forms [53,54]. In addition, simple ratios, such as C-reactive protein-to-albumin, or neutrophil-to-lymphocyte indices, have been validated as markers of systemic inflammation that may help distinguish infectious flares from autoimmune flares [36,37]. Future research should prioritize the validation of such biomarkers and indices in cohorts where diagnostic mimicry is frequent. Integrating these markers into operational definitions could improve the specificity of surveillance systems and reduce the epidemiological noise caused by infectious–autoimmune overlap.

## 6. Omission of Autoimmune Diseases from Epidemiological Surveillance Systems

A significant barrier to the study of mimicry between infectious and autoimmune diseases is that unfortunately, in Mexico, autoimmune diseases are not part of the group of conditions subject to mandatory epidemiological surveillance. Consequently, there are no operational definitions for entities, such as systemic lupus erythematosus, systemic vasculitis, multiple sclerosis, or juvenile idiopathic arthritis, which severely limits the system’s ability to identify, classify, and monitor them at the national level. In contrast, in developed countries, such as the United States, Canada, the United Kingdom, and Germany, national systems have already been established that allow for more structured surveillance of autoimmune diseases.

Although they do not cover all autoimmune diseases, these systems represent coordinated efforts between health institutions to integrate as many cases as possible and improve epidemiological knowledge of these conditions. For example, in the United States, the Centers for Disease Control and Prevention (CDC) coordinates the National Lupus Registry and actively collaborates with the National Institutes of Health (NIH) on population-based surveillance of autoimmune diseases [55]. In Canada, the Public Health Agency of Canada (PHAC) operates the Canadian Chronic Disease Surveillance System (CCDSS), which includes pathologies, such as rheumatoid arthritis [56]. In the United Kingdom, the National Congenital Anomaly and Rare Disease Registration Service (NCARDRS), part of the National Health System (NHS), is responsible for registering rare autoimmune diseases throughout the country [57].

Finally, in Germany, the Robert Koch Institute (RKI) plays a key role in coordinating epidemiological surveillance and promoting research on chronic and autoimmune diseases in the general population [58]. These examples show that, although surveillance of autoimmune diseases still faces challenges in terms of coverage, there are models that could be adapted or taken as a reference to generate future epidemiological surveillance of autoimmune diseases in such countries as Mexico. International experience shows that autoimmune diseases are more accurately tracked when surveillance systems include dedicated registries, standardized case definitions, and interoperable data. In the United States, the CDC–NIH National Lupus Registry captures cases through multiple sources and consistent definitions [55]. Canada’s CCDSS links administrative and clinical data to produce validated national estimates [56]. In the United Kingdom, NCARDRS registers rare autoimmune diseases and connects follow-up with policy decisions [57]. Germany’s RKI uses DEMIS, a nationwide electronic reporting platform with strict data-quality standards [58]. These models work by combining registry-based surveillance, harmonized definitions, and quality audits-elements that Mexico could adapt to strengthen autoimmune tracking. For policymakers, key lessons include adopting standardized case definitions, linking datasets with privacy safeguards, auditing data quality, establishing national registries for priority conditions, and ensuring that surveillance outputs inform resource allocation and equitable access to care [55,56,57,58].

The diagnostic overlap between infectious and autoimmune diseases not only creates challenges for clinicians but also weakens the quality of surveillance data. When syphilis or tuberculosis cases are mistakenly recorded as autoimmune conditions, the result is underreporting of infections and distortion of national incidence figures [28,29]. This reduces the reliability of surveillance systems and limits their ability to trigger timely interventions. Evidence from tuberculosis studies has shown that incomplete or inaccurate detection directly affects estimates of disease burden and, in turn, the prioritization of control programs [34]. Similar concerns have been raised for other conditions: when diagnostic uncertainty compromises data quality, surveillance loses its capacity to guide resource allocation and support evidence-based decision-making [3,29]. For this reason, updating case definitions to explicitly include infectious–autoimmune mimicry would not only improve diagnostic accuracy, but also help preserve the validity of surveillance indicators that are essential for public health planning.

Looking ahead, surveillance systems will need to evolve in ways that reflect the clinical reality of the overlap between infections and autoimmune diseases. One practical step is to develop structured differential diagnostic algorithms that guide both clinicians and surveillance officers in distinguishing infectious from autoimmune presentations. Such tools could help reduce misclassification and strengthen the reliability of reporting. Another important step is to broaden laboratory criteria, not only maintaining classical infectious tests, but also incorporating autoimmune markers, such as ANA, anti-dsDNA, or rheumatoid factor, together with molecular diagnostics for pathogens, like *T. pallidum* and *M. tuberculosis*. Bringing these elements into surveillance protocols—supported by training, pilot evaluations, and feedback loops—would create a more integrated framework, one that bridges clinical practice with immunological and molecular data. In the long term, this approach would allow surveillance to better capture the complexity of infectious–autoimmune mimicry and provide clearer guidance for policy and resource allocation.

## 7. Conclusions

The clinical and molecular mimicry between infectious and autoimmune diseases makes it necessary to question traditional epidemiological surveillance models, which tend to classify nosological entities without considering diagnostic overlaps. Therefore, it is necessary that epidemiological surveillance should be modified towards a combined syndromic and etiological approach, which allows a more accurate discrimination between infectious and autoimmune entities, particularly in clinically ambiguous contexts. This is particularly true in clinically ambiguous contexts. New molecular diagnostic technologies undoubtedly provide crucial information in discerning pathologies of an infectious nature. Finally, it is necessary to make autoimmune diseases visible within the national epidemiological surveillance system, as their exclusion is a limiting factor in the knowledge of the national profile of some of the most important diseases worldwide.

## Figures and Tables

**Table 1:** Main clinical manifestations of syphilis and tuberculosis that can lead to misdiagnosis by mimicking autoimmune diseases.

Infectious Entity	Mimetic Clinical Manifestations	Autoimmune Diseases That Can Mimic	References
Secondary syphilis	Rash on palms and soles Lymphadenopathy Patchy alopecia Migratory arthralgia Headache, lymphocytic meningitis Raynaud’s phenomenon	Rheumatoid arthritis Systemic lupus erythematosus Systemic vasculitis Sjögrens syndrome Systemic sclerosis	[30,31,32]
Extrapulmonary tuberculosis	Fever of unknown origin Migratory or monoarticular arthritis Sacroiliitis Persistent lymphadenopathy Non-specific skin lesions Lymphocytic meningitis Asthenia, weight loss, night sweats	Spondyloarthropathies Sarcoidosis Systemic lupus erythematosus Autoimmune diseases with meningeal involvement	[33,34]
Both (syphilis and TB)	Chronic anemia Asthenia, weight loss Night sweats Elevated ESR and CRP Lymphocytic pleocytosis in CSF Hypoalbuminemia	Systemic lupus erythematosus Multisystem inflammatory diseases	[35,36,37]

## Data Availability

Not applicable.
